# Physicochemical Characterization and Prospecting Biological Activity of Some Authentic Transylvanian Essential Oils: Lavender, Sage and Basil

**DOI:** 10.3390/metabo12100962

**Published:** 2022-10-11

**Authors:** Dan Vârban, Marius Zăhan, Carmen Rodica Pop, Sonia Socaci, Răzvan Ștefan, Ioana Crișan, Loredana Elena Bota, Ileana Miclea, Adriana Sebastiana Muscă, Alexandru Marius Deac, Rodica Vârban

**Affiliations:** 1 Faculty of Agriculture, University of Agricultural Sciences and Veterinary Medicine of Cluj-Napoca, Calea Mănăștur Street No. 3–5, 400372 Cluj-Napoca, Romania; 2 Faculty of Animal Science and Biotechnology, University of Agricultural Sciences and Veterinary Medicine of Cluj-Napoca, Calea Mănăștur Street No. 3–5, 400372 Cluj-Napoca, Romania; 3 Faculty of Food Science and Technology, University of Agricultural Sciences and Veterinary Medicine of Cluj-Napoca, Calea Florești No. 64, 400509 Cluj-Napoca, Romania; 4 Faculty of Veterinary Medicine, University of Agricultural Sciences and Veterinary Medicine of Cluj-Napoca, Calea Mănăștur Street No. 3–5, 400372 Cluj-Napoca, Romania; 5 Department of Biophysics, Life Sciences Institute “King Michael I of Romania”, University of Agricultural Sciences and Veterinary Medicine of Cluj-Napoca, Calea Mănăștur Street No. 3–5, 400372 Cluj-Napoca, Romania; 6 Agro-Botanical Garden (CLA), University of Agricultural Sciences and Veterinary Medicine of Cluj-Napoca, Calea Mănăștur Street No. 3–5, 400372 Cluj-Napoca, Romania

**Keywords:** quality, volatile, cancer cell line, pathogenic bacteria, secondary metabolites, analysis

## Abstract

Essential oils are a category of agro-based industrial products experiencing increasing demand. In this research, three essential oils obtained by steam distillation from lavender, sage and basil plants cultivated in temperate continental conditions of Transylvania were investigated for chemical composition, physical characteristics and biological activity (antimicrobial and cytotoxic effect on cancer cell lines). The number of identified compounds varied: 38 for lavender, 29 for sage essential oil and 41 for basil. The volatile profile was dominated by terpenes and terpenoids (>80%). Major components were beta-linalool and linalool acetate in lavender essential oil; thujones and camphor in sage essential oil; beta-linalool, thujone, camphor and eucalyptol in basil essential oil. Refractive index of the essential oils was lowest for lavender and highest for sage. Antibacterial activity was strongest for basil, moderate for lavender and weakest for sage essential oil. The most active on both colon adenocarcinoma (Caco-2) and ovary carcinoma (A2780) was sage essential oil.

## 1. Introduction

Essential oils (EOs) represent an important group of agro-based industrial products with wide utilization in food flavorings, pharmaceutical, agriculture, wellness, fragrance and cosmetics industries [1,2,3,4]. EOs have a long history of traditional use [5,6]. The European EO market experienced growth, because leading flavoring and fragrance manufacturers are located here. The European Union (EU) reported a 41% increase of EO production over the course of a single decade, while also presenting a doubling in value. Under these conditions, many EU countries increased their EO production [1].

EOs consist of a complex and diverse mixture of chemical constituents with low molecular weight. The aroma profile of EO can be given by a dominant constituent or a mixture of constituents depending on botanical identity [7]. Among the biological activities of interest for EOs are antioxidant, anti-inflammatory and anticarcinogenic activity, because these are responsible for the health-promoting properties most sought by people. In addition, given the rise in resistance of pathogenic microorganisms to common medicine, the antimicrobial activity is also of high interest [3,5].

There is a multitude of factors influencing EO characteristics, such as age of the plant [1], genotype and plant organ [5], time of harvest [7,8] and various environmental [7] and other agri-factors [9]. In addition, given the high demand, EO adulteration is not uncommon across supply chains [10,11]. Regardless of the destination of use, the variables of various factors could play a role on the final quality parameters of EOs, but their impact is important when it is related to the effectiveness and safety.

**Figure 1:**
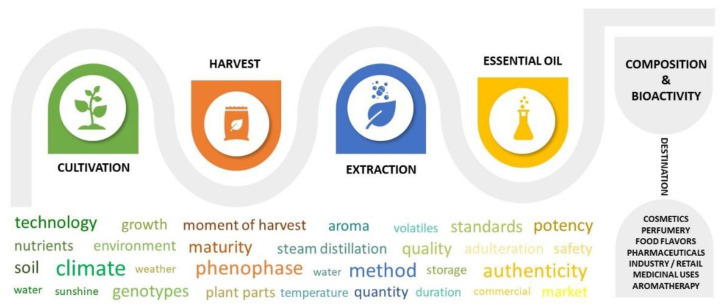
Production chain timeline of EO and factors that can potentially exercise cascading influence on the composition and bioactivity of essential oils (original).

The timeline of EOs from production to commercialization is a temporal sequence of interconnected links that concerns particularities related to cultivation, harvesting or extraction. These are potential contributors impressing an influence on quality and properties of EOs, as proposed in Figure 1.

Due to increasing demand, there is a need for transparency across the production chain timeline of EOs as a measure of green responsibility that can build robust knowledge bridging EO use with quality parameters. To date, there is stringent need to complete the knowledge base at an international level on the quality parameters of EOs in traceability systems. This requires that compelling information should be available on the identity of vegetal material sourced from given regions and obtained under known cultivation conditions, in the research publications. This could help researchers and industries to recognize and explain patterns of variation regarding certain physical–chemical as well as bioactivity parameters of EOs, that due to scarce or incomplete data cannot be achieved. Considering the lack of reports on quality of EOs from aromatic crops of Romania, this paper provides information on quality parameters for EOs, that could serve as reference for some physicochemical parameters and biological activity of EOs obtained in this region as well as a comparison base for the parameters of EOs from other regions.

The family Lamiaceae comprises herbs and shrubs, many having high economic importance [12,13,14,15], such as mint, lavender, thyme, sage, oregano, rosemary and basil [16], that find uses in agrochemical, pharmaceutical, food and cosmetic industries [1,17]. The plants present short glandular trichomes over the aerial organs [13,18], that can reach high abundance on certain plant parts depending on the species [13]. These plant epidermal structures contain aromatic ethereal oils [13]. Plants produce EOs as secondary metabolites playing diverse roles in their life [7]. In Table 1 there are presented particularities of the three species studied.

The aim of this research was to investigate some quality parameters of EOs for three frequently cultivated Lamiaceae species in Romania (Table 1), that could serve as orientation points for the potency of some EOs obtained locally. To reach this aim, four objectives were defined:

- evaluation of chemical composition of EOs,
- description of some physical parameters,
- evaluation of microbiological activity,
- evaluation of inhibitory activity on cancer cell lines.

The research was conducted in order to assess the potential of some medical and paramedical uses of EOs obtained locally, and determine the relationship between their composition and the bioactivity.

## 2. Materials and Methods

### 2.1. Location and Climate

The experimental field was located within Agro-Botanical Garden UASVM Cluj-Napoca, located at latitude 46°45′36″ N; longitude 23°34′24″ E; elevation (AMSL) 380–430 m. Mean annual temperature is 8.1 °C, and multiannual average sum of precipitation is 635 mm [32]. The local climate is transitional temperate–continental with oceanic influence and according to the Köppen–Geiger system is classified as warm humid continental climate (Dfb) [33]. Analysis for topsoil was conducted at an authorized laboratory—The Office for Agrochemical Studies in Alba County, Romania (Table 2).

### 2.2. Biological Material and Cultivation

This research focused on three aromatic species (two perennials and one annual) from the family Lamiaceae: lavender (*Lavandula angustifolia* cv. Sevstopolis), sage (*Salvia officinalis*) and sweet basil (*Ocimum basilicum*). Vouchers were deposited at the Herbarium of Agro-Botanical Garden of UASVM Cluj-Napoca (CLA), numbers 30302–30304. 

**Figure 2:**
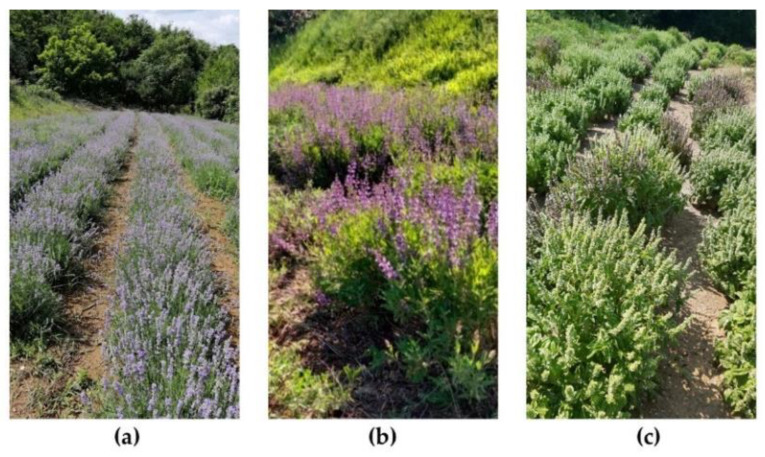
The experimental crops of lavender (**a**), sage (**b**) and basil (**c**) (original).

The lavender crop and the sage crop were established by seedlings in the year 2016, and therefore at the time of harvesting material for this experiment, the crops were 5 years old. The basil crop was established in the year 2021 by seedlings planted in the field in the month of May. The lavender crop was planted at 100 cm distance between rows and 50 cm between plants per row. The sage crop was planted at distances of 70 cm between rows and 30 cm between plants per row. The basil crop was planted at 70 cm distance between rows and 30 cm between plants per row. These distances are considered optimal for ensuring adequate nutrition space for these crops according to standard technology from the literature [16]. The size of the experimental field was 1500 m^2^. There was no fertilization applied, mechanical weed control was performed regularly. Harvesting was conducted by hand, at full flowering stage: June 2021 for sage, and July 2021 for lavender and basil. All the technological stages starting with the planting of the crop, harvesting and the EO extraction were conducted at UASVM Cluj-Napoca. Aspects from the field are presented in Figure 2.

### 2.3. Essential Oil Extraction Method

Essential oil extraction was achieved from the fresh harvested plant material. The essential oil extraction was obtained by distillation using extractor with a capacity of 100 L (model E0141, Albrigi In Herba, Stallavena, Italy). The fresh plant material was placed in the distillation unit, and water was added at the base. When the heated water temperature reached 80–85 °C, the steam rose through the plant material, mobilizing the essential oil from the plant tissue that was separated into a collector. The entire process for one lot of plant material lasted about 3 h, and during this time the temperature was maintained constantly at 85 °C. The extracted essential oil was packed in brown glass vials of 10 mL, sealed and stored in a dark and cool place.

### 2.4. GC-MS Qualitative Volatile Profile of Essential Oils

The volatile fingerprint of the essential oil samples was assessed by the gas chromatography coupled with mass spectrometry technique (GC-MS) using a GC-MS (QP-2010 model, Shimadzu Scientific Instruments, Kyoto, Japan) equipped with a Combi-PAL AOC-5000 autosampler (CTC Analytics, Zwingen, Switzerland). An aliquot from each essential oil sample was diluted in hexane and 1 μL injected in the GC-MS at a split ratio of 1:100. The separation of the volatile constituents was performed on a capillary column (ZB-5 ms, 30 m × 0.25 mm i.d. × 0.25 µm thickness, Phenomenex, Torrance, CA, USA) using the following temperature program: from 50 °C (maintained for 2 min) the temperature was increased to 160 °C at a rate of 4°/min and then to 250 °C with 15°/min and held for 10 min. The carrier gas was helium at a constant flow of 1 mL/min. The temperature for injector, ionic source and interface was set at 250 °C. The detection was performed on a quadrupole mass spectrometer operating in full scan (40–500 m/z), with electron impact (EI) as ion source at an ionization energy of 70 eV. The tentative identification of the volatile compounds was achieved by comparing their recorded mass spectra and the fragmentation patterns with those from the software’s NIST27 and NIST147 mass spectra libraries (considering a minimum similarity of 85%). The relative percentage of each compound was estimated as a fraction of its integrated ion area from the total ion chromatogram (TIC) area (100%).

### 2.5. Physical Parameters of EOs

#### 2.5.1. FT-IR Assay

Fourier transform infrared spectroscopy (FT-IR) analysis of the EO samples was conducted at the spectroscopy laboratory of the Life Sciences Institute “King Michael I of Romania” in Cluj-Napoca. Pellets were obtained from 200 mg calcined KBr using a Specac hydraulic press at a pressure of 10 tons for two minutes. The resulting vitrified pellets were transparent. A quantity of 2 μL EOs was used per pellet [35]. Immediately after removing the pellet from pellet discs, in the middle of the pellet was applied 2 μL of essential oil with the mechanical pipette, then the drop was slightly smeared and immediately the sample placed in the Fourier transform infrared spectrometer (Jasco FT/IR 4100). Measurements were conducted at the scanning range of 4000–350 cm^−1^ and resolution 4.0 cm^−1^, with accumulation of 256. Raw spectra received five corrections for CO_2_ and five corrections for H_2_O using Spectra Manager software. Then, spectra were exported to Origin software for peak readings. Interpretation was based on tentative assignment to bands according to data from the literature.

#### 2.5.2. Refractive Index

Refractive index analysis for the EOs of the three aromatic species was conducted at the Biophysics Laboratory of UASVM Cluj-Napoca, using an Optika refractometer with horizontal prism. Refractive index of the EOs as well as changes in refractive index for essential oils following mixing into a vegetable oil were investigated. The vegetable oil used was commercial grade canola oil (CO), produced locally (Luna Solai, Luncani, Romania). Readings were performed for the following concentrations: 100% EO, 50% EO + 50% CO, 40% EO + 60% CO, 30% EO + 70% CO, 20% EO + 80% CO, 10% EO + 90% CO, 100% CO. Sample drops were placed with a pipette abundantly on the horizontal prism, then prisms were locked and the reading was performed according to the instructions manual.

### 2.6. Inhibitory Activity of EO against Bacterial Strains

The following microorganisms were tested: *Staphylococcus aureus* ATCC 6538P, *Escherichia coli* ATCC 25922, *Salmonella enteritidis* ATCC 13076 and *Listeria monocytogenes* ATCC 19114. Each strain was grown in a test tube containing 10 mL sterile nutrient broth (Oxoid Ltd., Basingstoke, Hampshire, UK) at 37 °C for 24 h. The purity of the inoculum was confirmed by microscopic examination of the Gram-stained smear. A loopful of inoculum was transferred to selective medium: Baird-Parker agar base supplemented with egg yolk tellurite emulsion for *S. aureus*, TBX agar for *E. coli*, XLD agar for *Salmonella enteritidis* (Oxoid Ltd., Basingstoke, Hampshire, UK) and Palcam agar (Oxoid Ltd., Basingstoke, Hampshire, UK) for *Listeria monocytogenes*. Plates were incubated for 24 h at 37 °C. Bacterial morphology was confirmed by optical microscopy. Several colonies were transferred to sterile saline solution (8.5 g/L), and adjusted to match the turbidity of McFarland 0.5 standard (1.5 × 10^8^ CFU/mL) [36,37].

#### 2.6.1. Determination of the Minimum Inhibitory Concentration (MIC)

The MIC was determined using the resazurin microtiter plate-based antibacterial assay. Stock solutions of the essential oils were prepared in eight parts 50% ethanol by mixing with one part Tween 80 [37]. Into the first well of a 96-well microtiter plate, 100 µL sterile nutrient broth and 100 µL sample were added. Serial 11-fold dilutions were performed by transferring 100 µL from well to well (on row). From the last well of the row, 100 µL was discarded. To each well, 10 µL of inoculum (1.5 × 10^8^ CFU/mL) was added. Gentamicin (0.04 mg/mL in saline solution) was used as a positive control. For the negative control, a mixture with one part of the saline solution, eight parts of 50% ethanol and one part of Tween 80 was used. Microplates were incubated for 20–22 h at 37 °C. To each well, 20 µL resazurin aqueous solution (0.2 mg/mL) was added. Microplates were incubated for 2 h at 37 °C. The concentration that completely inhibited bacterial growth (MIC) was the concentration at which the blue color did not change into pink. Three replicates were run for each sample.

#### 2.6.2. Determination of the Minimum Bactericidal Concentration (MBC)

MBC was determined by plating a 10 μL aliquot on solid culture Mueller–Hinton medium (Oxoid Ltd., Basingstoke, Hampshire, UK) from the last 4 wells that showed inhibition of bacterial growth in the MIC testing. The plates were incubated for 24 h at 37 °C. The lowest concentration that prevented the growth of bacteria (no colonies on the plate) was considered the MBC. Three different biological replicates were performed for each plate [38].

### 2.7. Cytotoxicity Screening of EO in Cancer Cell Lines

The experiments were carried out on two human tumor cell lines: Caco-2 (colon adenocarcinoma, ATCC HTB-37) and A2780 (ovary carcinoma, ECACC 93112519). Caco-2 was cultured in Eagle’s minimum essential medium (MEM) containing 2 mM L-glutamine, 1 mM sodium pyruvate, 1% (*v*/*v*) NEAA, supplemented with 10% (*v*/*v*) fetal bovine serum (FBS) and without antibiotics, while A2780 was cultured in RPMI-1640 supplemented with 2 mM L-glutamine, 10% FBS and without antibiotics, in an atmosphere of 5% CO_2_ in air, 95% relative humidity and 37 °C (Memmert, INCO2). At approx. 80% confluence, cell lines were detached using 0.25% (*w*/*v*) trypsin–0.53 mM EDTA solution and were seeded in 96-well microplates at a concentration of 5 × 10^4^ cells per well in 200 μL culture medium. After 24 h, 0.001, 0.002, 0.004, 0.008, 0.016, 0.032 and 0.064% (*v*/*v*) for each EO (lavender, sage and basil) were added to the culture medium and cells were incubated for the next 24 h under the same conditions. Tween 20 was used as a solvent for EO (10%) and the cytotoxic activity of Tween 20 was also tested at the maximum concentration of 0.0064%. Working dilutions were freshly prepared on the day of testing. At the end of incubation time, cells of each well were examined in contrast phase microscopy (Olympus IX51). The cytotoxicity assay was assessed by using 3-(4,5-dimethyl-2-thiazolyl)-2,5-diphenyl-2H-terazolium bromide reagent (MTT). After a PBS washing step, cells were incubated with 150 μL/well MTT solution (5 mg/mL) for 1 h at 37 °C. The resulting formazan crystals were dissolved in 150 μL/well dimethyl sulfoxide (DMSO). The absorbance values were measured using wavelengths of 550 nm and 630 nm with an HT BioTek Synergy microplate reader (BioTek Instruments, Venusky, VT, USA). Cell viability was expressed as percentage of control (cells incubated in normal medium only). All experiments were conducted in triplicate.

### 2.8. Statistical Analysis for Microbiological Activity

Data were reported as average mean ± standard deviation (SD) for triplicate determinations. The ANOVA analysis of variance was used to compare the average mean values, using SPSS 19.0 for statistical analysis (IBM, New York, NY, USA) and Tukey’s honestly significant difference (HSD) test with a confidence interval of 95% or 99%. A *p*-value below 0.05 was considered statistically significant.

## 3. Results

### 3.1. GC-MS Results

For the lavender essential oil, 40 compounds were separated and of these 38 identified based on their mass spectra (29 terpenoids, 2 alcohols, 1 ketones and 5 esters, 1 hydrocarbon). The terpenes and terpenoids represented 94.41% of the volatile profile. The major volatile compounds were beta-linalool (30.91%) and linalool acetate (28.75%), followed by caryophyllene (5.66%) and beta-farnesene (6.45%) while camphor was identified only in a very small percentage (0.18%). Beta-linalool and linalool acetate were also the main compounds identified in the lavender essential oil from Middle-Friuli Venezia Giulia [39] together with the low amount of camphor. The lavender essential oils with this type of volatile profile are considered of highest quality (Table 3).

In the case of the sage essential oil, oxygenated monoterpenes are the main chemical group with thujones (39.72%) and camphor (16.17%) (Table 3) being the major compounds, in accordance with other studies in the field [40,41]. Scientific literature suggested that the ratio between camphor and thujones can be consider a quality parameter for the *Salvia officinalis* essential oil, with thujone concentration up to half of the total and camphor not less than 20%. Caution is required on the part of the public when using EOs. According to Cvetkovikj et al. [41], an important aspect regarding the safety of *Salvia* sp. EO is related to the toxicity of cis- and beta-thujone and camphor (an issue that is addressed in the literature [42,43] with their content limited).

In the case of basil essential oil, there were 53 separated compounds of which 41 were identified. The terpenic compounds represented >80% of the total volatile profile with beta-linalool (26.27%), thujone (14.84%), camphor (5.39%) and eucalyptol (5.22%) as the main representatives (Table 3). There is a wide variety of basil subspecies, with their chemical composition, including the volatile profile, being dependent on intrinsic (genetics) or extrinsic factors such as season and conditions of cultivation [44]. Nonetheless, the monoterpenes are the overwhelming chemical group in all basil species, in agreement with the findings of other authors [45].

### 3.2. Physical Characteristics of EOs

#### 3.2.1. FT-IR Results

Two spectral ranges were identified and examined. First, between 400–2000 cm^−1^, where most particularities were observed, and a second range between 2000–4000 cm^−1^ where the only differences between spectra were due to differences in band absorbance.

**Figure 3:**
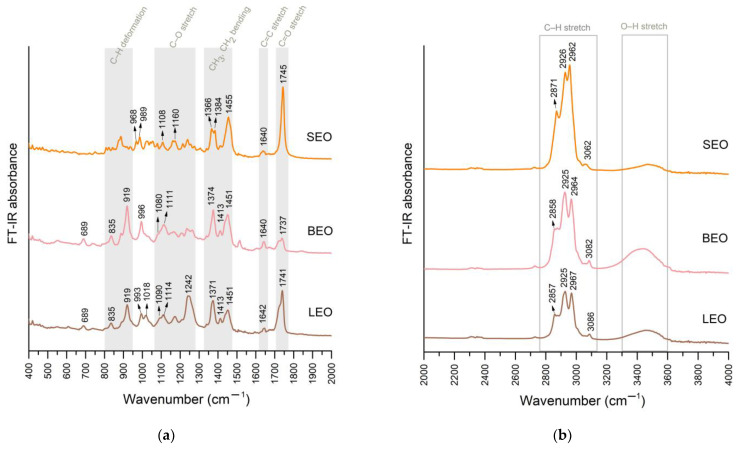
FT-IR spectra of EOs, in the regions 400–2000 cm^−1^ (**a**) and 2000–4000 cm^−1^ (**b**) (LEO—lavender essential oil; SEO—sage essential oil; BEO—basil essential oil).

FT-IR spectra of lavender and basil EOs presented peaks at 689 and 835 cm^−1^, that could be assigned to the C–H molecular bond vibrations, as attributed in previous studies on basil and lavender EOs [46]. A study on lavender EO attributed these deformation vibrations to molecular bonds from the structure of linalool [47]. In the sage EO of this study, the first peak mentioned above was not present, while in the region 800–850 cm^−1^ the sage oil presented several peaks of low intensity compared to lavender and basil that presented just one distinctive peak (Figure 3a). The peak observed at 919 cm^−1^ only in the EO samples of lavender and basil could be attributed to C–H deformation vibrations from the structure of eucalyptol and linalool, according to a study on lavender EO [47]. Eucalyptol was present in all three EOs, but in a higher concentration in basil (Table 3), that could also explain the higher absorbance observed (Figure 3a).

At around 1000 cm^−1^, all three EOs presented a band that displayed only one peak in basil and double peaks in lavender and sage. In the region 1015–1095 cm^−1^, only sage EOs displayed several peaks of low intensity that were not present in the other two EOs. A study on sage EO from Albania suggested that two or more bands occurring from 1070–1210 cm^−1^ could be attributed to ethers (saturated, branched) [48]. Sage EO also presented peaks at 1108 and 1160 cm^−1^ that could be assigned to terpenes with tertiary and secondary alcoholic functions [35]. Lavender and basil also presented a peak of higher intensity than observed for sage at 1111 and 1114 cm^−1^, respectively, that can be assigned to C–O stretching vibrations [46]. This peak identified in basil and lavender also displayed a shoulder at 1080 and 1090 cm^−1^, respectively, that was not present in sage. Sage EO displayed a distinctive peak of low intensity at 1079 cm^−1^ that was not observed in the other two spectra. A study from France on lavender and lavandin EOs assigned the bands at 1079–1082 cm^−1^ to camphor [47]. Considering that camphor had the highest concentration in sage EO compared to the other two (Table 3), the peak at 1079 cm^−1^ could be attributed to camphor. Study on EOs of several Lamiaceae species indicated that the area between 1100 and 1300 cm^−1^ corresponds to stretching vibrations of the C–O group, while the O–C–O band originating from primary alcohols appears in the region from 1020 and 1100 cm^−1^ [49].

From 1237–1242 cm^−1^, all three EOs presented a peak (Figure 3a) that could be assigned to C–O stretching vibrations [46]. Lavender and basil presented a single sharp peak at 1371 and 1374 cm^−1^, respectively, that could be attributed to C–H bending vibration [46]. By comparison, in the same region, sage EO displayed double peaks: 1366 and 1384 cm^−1^. The FT-IR absorption band at 1375 cm^−1^ of sage EO from Italy was assigned to the symmetrical bending of the methyl C–H bonds [35]. FT-IR spectra of sage EO from Albania displayed a band at 1366–1461 cm^−1^ assigned to symmetric, asymmetric bending of CH_3_ and CH_2_ [48]. Another study on lavender and lavandin showed that weak bands from 1357–1390 cm^−1^ and 1412–1483 cm^−1^ could be attributed to CH deformation vibration of methyl and methylene groups [47]. All three EOs displayed a low intensity peak at 1413 cm^−1^, followed by a strong band with a single peak at 1455 cm^−1^ for sage, 1451 cm^−1^ for lavender and basil. A study on lavender and basil attributed the band of this region to C–H bending vibration [46]. A study on several Lamiaceae EOs, including lavender and sage, through ATR-FTIR, attributed the peak at around 1420 cm^−1^ to the =CH_2_ in-plane deformation. ATR-FTIR investigation of some Lamiaceae EO concentrates indicated that the peak occurring at ~1450 cm^−1^ is a result of an overlap of CH_2_ deformation vibration with asymmetrical CH_3_ deformation vibration. The intensity of this peak was shown to be dependent on and proportional to the number of CH_2_ and CH_3_ functional groups found in the sample. Furthermore, the =CH_2_ in-plane deformation vibration was not found as a separate band near 1410 cm^−1^, because it could be concealed under the −CH_3_ and =CH_2_ absorption bands. The cited authors further inferred that higher intensity at 1330–1410 cm^−1^ for some terpenes could be due to the presence of a =CH_2_ group [49]. FT-IR investigation of sage EO from Italy identified CH_2_ bending vibrations detectable at 1458 cm^−1^ [35].

All three species presented a weak peak at 1640 cm^−1^ in sage and basil and at 1642 cm^−1^ in lavender EO. A study on lavender and basil EOs attributed this to the C=O stretching vibration [46]. Another study on lavender attributed this signal to linalool and lavandulyl acetate [47]. A study on EOs of several Lamiaceae species including lavender and sage through the ATR-FTIR method showed that low-intensity peaks from 1635–1650 cm^−1^ were attributed to RHC=CH_2_ vibration of linalool and linalool acetate [49]. In this study, the band was present in all three samples but intensity was higher for basil EO, that also presented a higher linalool concentration compared to the other two. As lavandulyl acetate concentration was in the lower range, the contribution to this peak did not cause increased absorbance in the case of lavender EO, compared to the other two. The band at 1645 cm^−1^ for FT-IR spectra of sage EO from Italy assigned this signal to C=C stretching mode of a vinylidene double bond such as the ones from camphene [35]. The FT-IR spectra of sage EO from Albania also displayed a peak at 1640 cm^−1^ attributed to monoterpenes, in this case pinene [48]. In this study, both camphene and pinene were found in higher concentrations in sage EO (Table 3), and could explain the presence of this peak, in spite the fact that linalool was found in very low quantity and lavandulyl acetate was not present, as it is not specific to this plant.

The next band with strong intensity in lavender and sage and medium intensity in basil was located at: 1737 cm^−1^ in basil, 1741 cm^−1^ in lavender and 1745 cm^−1^ in sage, attributed in all three cases to C=C stretching vibration [35,46,47]. An investigation of commercially sourced sage EO by ATR-FTIR, evidence of a spectrum with a peak at 1745 cm^−1^ attributed to carbonyl stretching from α-thujone and camphor was found [49]. Similarly, the FT-IR spectra of sage EO from Albania displayed a peak attributed to camphor and thujone at 1734 cm^−1^ [48]. Considering that sage EO presented the highest concentration of camphor, this might explain the higher intensity of this peak compared to the other two (Figure 3a). In lavender, this peak could also be attributed to C=O stretching vibration [46] of lavandulyl acetate [47], and the contribution of this vibration could have caused the second highest intensity of this band in lavender EO from this study.

From 2800–3000 cm^−1^, all three EOs displayed a strong band with three distinctive peaks (Figure 3b), situated for sage at 2871, 2926 and 2962 cm^−1^, for lavender at 2857, 2925 and 2967 cm^−1^ and for basil at 2858, 2925 and 2964 cm^−1^ and attributed to –CH_2_ stretching [46]. FT-IR spectra of sage EO from Albania attributed the bands from the region 2849–2956 cm^−1^ to symmetric and asymmetric C-H stretches (CH_3_, CH_2_) [48]. FT-IR spectra of sage EO from Italy displayed peaks at 2872, 2927 and 2956 cm^−1^, attributed to alkane asymmetrical and symmetrical C-H stretches [35].

The broad band at 3400–3500 cm^−1^ in Lamiaceae family volatile extracts was attributed to the content of phenolics (and flavonoids), that are more abundant in this medicinal plant family compared to others such as Asteraceae [49]. The signal of this spectral region is due to O-H stretching, H-bonded for alcohol and phenol [35]. In this current study, the band was more intense for basil, followed by lavender and, lastly, by sage.

#### 3.2.2. Refractive Index

**Figure 4:**
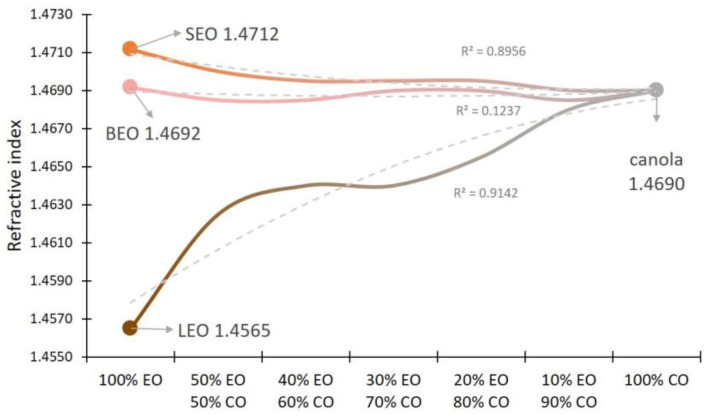
Refractive index of EOs under dilution with canola oil (CO) and logarithmic trendline. (LEO—lavender essential oil; SEO—sage essential oil; BEO—basil essential oil).

Lavender EO presented the lowest refractive index out of the three species (1.4565 ± 0.0005 SD), and sage EO the highest (1.4712 ± 0.0003 SD). Canola oil presented a refractive index of 1.4690, which was the closest to the refractive index of basil EO (1.4692 ± 0.0003 SD). With incremental dilution with the vegetable oil, the optical properties changed and the refractive index shifted (Figure 4), with a trend towards increased similarity to the refractive index of the dominant component (the canola oil).

### 3.3. Inhibitory Activity of EO against Bacterial Strains

The bacterial strains used in the present study are more or less susceptible to each essential oil. The results related that the tested essential oils were characterized by varied antimicrobial activity. Gram-negative bacteria are more resistant to sage than to lavender and basil essential oils; however, sage EO manifests similar behaviors against *Staphylococcus aureus* ATCC 6538P. Table 4 summarizes the results of the resazurin microtiter plate-based antibacterial assay.

The results obtained show that basil EO is the most effective against *Escherichia coli* ATCC 25922, followed by *Salmonella enteritidis* ATCC 13076 and by *Staphylococcus aureus* ATCC 6538P. This result is in accordance with that found by Gaio et al. [50]. The EO of sage reveals the lowest MIC against *Escherichia coli* ATCC 25922, followed by *Listeria monocytogenes* ATCC 19114; lavender EO manifests similar behaviors against *Listeria monocytogenes* ATCC 19114. The results confirm that the antibacterial activity of basil EO is strong, that of lavender EO is moderate and that of sage EO is weak.

### 3.4. Cytotoxicity of EO in Cancer Cell Lines

**Figure 5:**
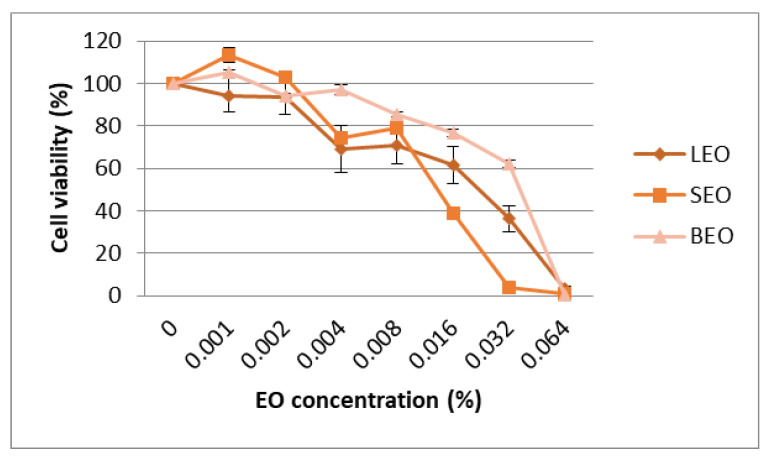
Cytotoxic effect of the essential oils on the Caco-2 cell line assessed in the MTT test (LEO—lavender essential oil; SEO—sage essential oil; BEO—basil essential oil).

**Figure 6:**
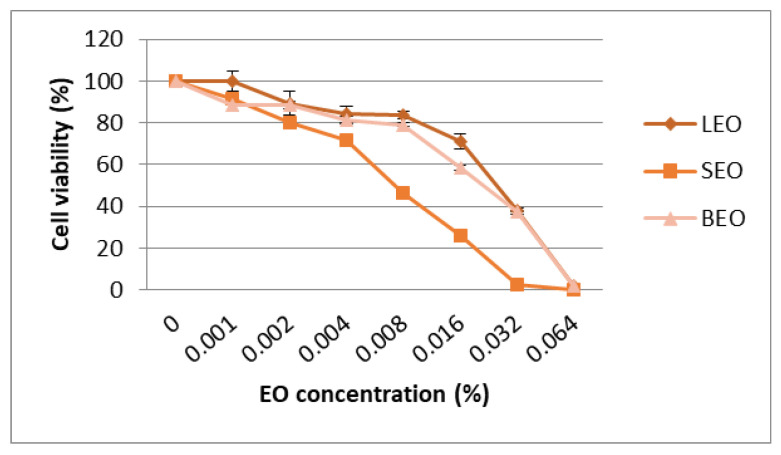
Cytotoxic effect of the essential oils on the A2780 cell line assessed in the MTT test (LEO—lavender essential oil; SEO—sage essential oil; BEO—basil essential oil).

The cytotoxic effect of the lavender, sage and basil essential oils was evaluated using the MTT assay and the results were expressed as percentage of the control. The results of cytotoxicity on the Caco-2 cell line are shown in Figure 5. The IC50 values (median inhibitory concentrations that cause approximately 50% cell death) were 0.027% for lavender EO, 0.024% for sage EO and 0.034% for basil EO (Figure S1). Cytotoxic activities of EO on the A2780 cell line are shown in Figure 6. The IC50 values were 0.030% for lavender EO, 0.017% for sage EO and 0.027% for basil EO. The most active on both colon adenocarcinoma (Caco-2) and ovary carcinoma (A2780) was sage EO, while less cytotoxic activity was provided by basil EO on Caco-2 and lavender EO on A2780 cell culture (Figure S2). Morphological analysis of cells showed a large number of shrunken and detached cells after exposure to higher concentrations of EO. This process was more obvious for the concentrations of 0.016% and 0.032% of sage EO compared to lavender EO or basil EO, in both cell lines (Figure 5 and Figure 6).

## 4. Discussion

Essential oils (EOs) are recognized for their health-promoting properties and having diverse applications in various industries and agriculture. EOs can vary strongly depending on geographical origin of the plant material [51], and references for particularities arising due to provenances across geographical locations are not well documented in the literature. Compliance with well-defined quality standards for the plant material destined for essential oil production could be a way to reduce the variability in quality. In this regard, good agricultural practices (GAPs) have been proposed as a possible approach for obtaining high-quality phytomedicine in the past [52]. One can infer that compliance with GAPs can also be of great importance for ensuring higher quality EOs.

Due to the high demand for and increasing use of EOs, regulations with the purpose to maintain a high standard of quality and safety remain of central importance. In the European Union, regulation of commercialized EOs falls under the incidence of Regulation EC Number 1907/2006 EC on “Registration, Evaluation, Authorization and Restriction of Chemicals” (REACH) currently in force [53], as well as a few other inherent regulations for some uses of EOs, such as flavorings in the food industry or their use in cosmetic products [1]. With the REACH regulation, the European Chemical Agency (ECHA) was established with the purpose to gather, manage and supervise the registration of substances by manufacturers and importers [54]. In addition, two entities, the European Federation of Essential Oils (EFEO) and the International Fragrance Association (IFRA), publish up-to-date guides on the substance identity of EOs and environmental assessment guidance on EOs [55].

Although authentication of EOs can be obtained by employing a variety of techniques such as sensory analysis and screening various chemical and physical parameters against standards [51], the adulteration of essential oils might be also shown by bioactivity, particularly when the destination of use is related to medical or paramedical purposes [56,57]. In this regard, it is proposed that a harmonization of benchmark standards for biological activity could be released for EOs destined for paramedical uses, to ensure safety and especially effectiveness.

Following the results of this study, it was determined that the three essential oils obtained from local crops reached high standards with regard to their composition, while the biological activity showed effectiveness with some variability between species attributed to their composition.

Physical parameters can also be powerful tools in the characterization of EOs. IR spectral particularities could be used for defining fingerprint regions for botanical identification or potential authentication. Investigation of 30 essential oils from several botanical families by ATR-FTIR revealed, based on PCA clustering, that the strongest influences on the principal components in the Lamiaceae family group EOs are the bands at around 842, 1375, 1450 cm^−1^ and the broad one at 3400–3500 cm^−1^ [49]. Research also shows that the hydrogen bond can cause changes in vibration frequency of essential oils that contain O-H and N-H bonds. The absorption band position given by valence vibration of OH bonds can be used to quantify the strength association through hydrogen bonding. By dilution with a solvent that does not participate in hydrogen bonding such as non-polar organic solvent, there occurs a narrow band range located at higher frequency characterized by the valence vibration of the OH unassociated bond [58]. Dilution of an essential oil with a non-polar organic solvent not participating in hydrogen bonding leads to significant differences in terms of the shape and intensity of the respective bands, particularly in the region from 1000–1300 cm^−1^ [49]. Optical physical parameters of EOs, such as refractive index, can serve in certain cases as qualitative diagnosis of EOs. In this study, it was shown that, particularly in the case of lavender oil, the dilution with a vegetable oil caused a steep shift in the refractive index.

Both lavender and basil EOs from this study presented as major components beta-linalool and linalool acetate. Linalool (3,7-dimethyl-1,6-octadien-3-ol) is an acyclic monoterpene tertiary alcohol with volatile flavors, that was extracted from more than 200 plants worldwide [59,60]. Linalool is one of the active constituents of many EOs. Studies regarding the antibacterial activity of linalool demonstrated antimicrobial activity against *L. monocytogenes* [60]. *Listeria monocytogenes* is an opportunistic foodborne pathogen which often shows resistance against antimicrobials and lavender EO. Aelenei et al. [61] studied the antibacterial activity of linalool against some Gram-positive (*Staphylococcus aureus*, *Staphylococcus epidermidis*) and Gram-negative bacteria (*Escherichia coli*) using broth microdilution assays. Adaszyńska-Skwirzyńska and Szczerbińska [62] demonstrated that the Gram-positive (*Staphylococcus aureus*) bacteria were the most vulnerable to the lavender essential oil. Additionally, the growth of strains such as *S. aureus*; *S. aureus* MRSA/ORSA; *C. albicans*; *E. coli*; *P. aeruginosa*; *S. pullorum; S. enteritidis; S. typhimurium* was inhibited by linalool. Relevant studies have reported that lavender essential oil of Moldovan origin showed good antibacterial activity against both non-pathogenic Gram-positive and Gram-negative bacteria (*B. subtilis* and *P. fluorescens*) [63]. Similarly, another study has shown mostly strong antibacterial activity of lavender essential oil against Gram-positive bacteria and against Gram-negative bacteria [64]. Although the antimicrobial properties of different essential oils have been recognized for a long time, the interest in this alternative antibacterial potential is relatively recent, with their efficacy on pathogenic microorganisms [50]. The basil essential oil presented a high antibacterial activity against *Staphylococcus aureus*, but a low activity against *Pseudomonas aeruginosa* [50]. The present investigation revealed that the Gram-positive bacteria tested were more resistant to basil essential oil than Gram-negative bacteria. This tendency is also observed by other authors [50,65,66]. The basil (*Ocimum basilicum* var. *purpureum*) EO extracted from air-dried plant material (aerial parts only) by hydro-distillation was quite active against *B. subtilis* [65]. The results of the resazurin microtiter plate-based antibacterial assay demonstrated that the basil EO exhibited potential activity against some representative food-borne pathogenic bacteria such as *S. aureus, L. monocytogenes*, *E. coli* and *S. enteritidis.* The tested sage essential oil was active against the *S. aureus* strain with an MIC value of 3.795 µL/mL. Similar results were obtained by Sienkiewicz et al. [67] for clary sage essential oil, which was active against *S. aureus* clinical strains with MIC values ranging from 3.75 µL/mL to 5.25 µL/mL. According to the results obtained by Ghavam et al. [68], the antibacterial activity against different microorganisms could be caused by different components of the oil, which depend on the different plant parts used for extraction, especially the leaves and flowers. Some research found that the antimicrobial activity of essential oils can be higher/lower than or closely related to the activity of the main components, which in turn is caused by different interactions between essential oil compounds. Usually, essential oils have higher antimicrobial activity than their major components, which suggests possible interactions between all constituents [69].

Biological activity of different types of lavender EOs was tested in vitro on a large number of cell types. A notable result was achieved by Prashar et al. [70] regarding the cytotoxic effect of lavender EO (*L. angustifolia*) on human skin cells (HMEC-1, HNDF and 153BR) at a 0.25% concentration. Kozics et al. [71] determined a value of IC50 of 0.043% on the human cell line HaCaT (normal keratinocytes). The HaCaT line was used by Miastkowska et al. [72] too, observing that in the case of commercial *L. angustifolia* EO the value of IC50 was lower (0.20%) than that of the in-house one (0.36%). In another study conducted by Niksic et al. [73], a significant antiproliferative activity of *L. angustifolia* EO on three cancer cell lines, MOLT-4 (acute lymphoblastic leukemia), MCF-7 (mammary adenocarcinoma) and NCI-H460 (pulmonary carcinoma), was reported. In the research of Zhao et al. [74], the IC50 value for *L. angustifolia* EO depended on the type of prostatic cell line: 0.037% for PC-3 and 0.199% for DU145.

Forty-eight-hour treatments with EO from aerial parts of *L. atriplicifolia* on LoVo (colorectal) and HepG2 (hepatocellular) cell lines also had cytotoxic and antiproliferative effects at a concentration of 10 µg/mL [75]. Even if most of the studies tried to identify the IC50 value of a certain lavender EO cytotoxicity effect, this parameter is not the only one studied. For example, Lopez et al. [76] studied the pharmacological mechanism of lavender EO on central nervous system targets, while Pandur et al. [77] explored the anti-inflammatory effects of *L. angustifolia* EO.

In vitro studies concerning anti-inflammatory and immunomodulatory effects have also been explored in the case of sage EO [78,79]. However, most of the in vitro studies focused on the cytotoxic and antitumor effects of *S. officinalis* EO on different cell types [80,81,82,83,84]. The growth-inhibitory and proapoptotic effects of eighteen sage EOs were evaluated in three human melanoma cell lines, A375, M14 and A2058 [82]. The results showed that the potential anticancer activity of the sage EO may be related to the active α- and β-thujone isomers associated with the synergism of other compounds such as camphor. Mohammed et al. [84] observed that the EO yields, compositions and biological activity levels of the fresh and differently timed and room-temperature dried herbs differed slightly. However, the EO obtained from two-week dried sage herbs exhibited better protection, as well as anticancer activities on the MCF-7, HepG-2 (hepatocellular carcinoma) and HeLa (cervical carcinoma) cell lines.

The cytotoxic effect of different fractions of *S. officinalis* EO was tested on the RAW264.7 (murine macrophage) and HCT-116 (colon cancer) lines [81], and the results showed a higher cytotoxic activity in the case of fractions that contained α-humulene compared with the fractions that contained trans-caryophyllene. In the case of certain bioactive components from S. libanotica EO, Itani et al. [85] observed that three of them (linalyl acetate, terpeniol and camphor) caused synergistic inhibition of the growth of two isogenic HCT-116 lines (human colon cancer cell lines; p53+/+ and p53−/−) but did not have any effect on growth of FHs74Int (normal human intestinal cell line). The type and the concentration of certain bioactive components are influenced by the plant source too. In this regard, the research of Kuźma et al. [86] points out the fact that the EO from in vitro regenerated plants of *S. sclarea* exhibited stronger cytotoxic action against NALM-6 (lymphoblastic cell line) in comparison with the essential oil from in vivo plants.

In an article published in 2022, Perna et al. [87] reviewed the anticancer ability of the extracts of various *Ocimum* sp. genotypes, including *O. basilicum*. Based on sixteen published studies, basil demonstrated important anticancer activities in in vivo and in vitro models, suggesting that it could act as a potential anticancer agent. The EO isolated from *O. basilicum* L. was analyzed by Mahmoud [88] in order to establish in vitro and in vivo anticancer activity using human promyelocytic leukemia cell lines (HL-60 and NB4) and Ehrlish ascites carcinoma cells (EACCs). In his study, basil EO showed cytotoxic effects with IC50 values of 78.9 μg/mL on HL-60 and 92.2 μg/mL on NB4. In another study, Aburjai et al. [89] concluded that the essential oils of *O. basilicum* leaf extract have significant anticancer activity on the U-87 MG (glioblastoma), MDA-MB-231 (breast cancer) and MCF-7cancer cell lines. Kathirvel and Ravi [90] observed that *O. basilicum* EOs obtained from plants collected from the Western Ghats area may belong to the methyl cinnamate and linalool chemotype and were shown to be active against the HeLa, HEp-2 and NIH 3T3 cancer cell lines. On the other hand, the study of Taie et al. [91] showed an increase in antioxidant and antitumor potential of the basil EO in the case of organic and bio-organic fertilized basil. These results indicated that organic and bio-organic fertilization can have a significant increasing effect on the antioxidant activity, anticancer activity, phenols, flavonoids and EO profile of *O. basilicum* plant extract.

All these studies demonstrate that in recent years there has been an intense preoccupation and concern regarding the most accurate identification of biological activity of EOs. Particularly, there is a growing interest in the effects on different normal or tumor cell types, that could be fundamental for not only external medical uses but also internal uses. The results obtained to date confirm a large number of factors that influence the cytotoxicity of these products, such as genus and species, method of extract action, chemical composition (influenced by soil, climate) and also the effect of cellular type upon their action. The activity against pathogenic bacteria suggests that effectiveness depends on the synergistic effect of the specific composition found in a given EO, with inhibitory activity dependent on botanical identity, and therefore on the specific composition of the EO.

## 5. Conclusions

This study investigated the physical and chemical composition as well as the preliminary biological activity of essential oils from three commonly cultivated species of the family Lamiaceae: lavender, sage and sweet basil. The EOs were obtained by steam distillation from plant material cultivated in climatic conditions of Transylvania, Romania.

Results showed that in all three species, terpenes and terpenoids dominated the volatile profile: 94.41% in lavender EO, 95.35% in sage EO and 81.31% in basil EO, with over 20 terpenes and terpenoids identified in each case. Based on the literature, the EOs obtained are of high quality.

Investigation of the physical properties of EOs through FT-IR identified signals of the main functional groups from the structure of various compounds of the EOs studied, suggesting that FT-IR spectra particularities might be used for defining fingerprint regions for botanical identification. Refractive index was lowest for lavender EO (1.4565), and highest for sage (1.4712), and changes occurred following dilution with a vegetable oil. Therefore, refractive index could be optimized to serve for the qualitative diagnosis in the authentication of EOs. Investigation of antibacterial activity showed that the effect of basil EO was strong, that of lavender EO was moderate and that of sage EO was weak.

Cytotoxicity of EOs on cancer cell lines showed that the most active on both colon adenocarcinoma (Caco-2) and ovary carcinoma (A2780) was sage EO, while less cytotoxic activity was exhibited by basil EO on Caco-2 and lavender EO on A2780 cell culture.

## Figures and Tables

**Table 1:** General overview on the three species studied.

Botanic Name	*Lavandula angustifolia* Mill.	*Salvia officinalis* L.	*Ocimum basilicum* L.
Common name	lavender	sage	basil
Origin/native range of the species	mountainous regions of the Mediterranean [19]	Middle East and Mediterranean [20]	tropical regions of the Old World [21,22]
Structures containing/ accumulating EO	capitate and peltate trichomes [23]	capitate, peltate trichomes [24,25] and ambrate resinous droplets [25]	capitate and peltate trichomes [26]
Location of highest abundance in EO storage structures	flower calyx [8,23,27]	both sides of the leaf [17,25]	abaxial leaf surface [28]
Harvested plant part and optimal time for EO extraction	inflorescence/upper plant part at full flowering stage [23,27] after midday [29]	leaves and shoots at full flowering, in the evening [30]	herbs at bud flowering stage [31]

**Table 2:** Soil characteristics of the experimental field.

Parameter	Result	Interpretation ^1^
Soil reaction (pH)	7.50	slightly alkaline
Humus %	3.30	middle range
Total nitrogen (Nt %)	0.155	middle range
Phosphorus (P ppm)	22.0	middle range for field crops
Potassium (K ppm)	185	good for field crops

^1^ Compared to thresholds [34].

**Table 3:** Volatile profile of the essential oils from the three Lamiaceae species.

Category	Compound	Concentration (% from Total Peak Area)		
		LEO	BEO	SEO
terpenes/ terpenoids	(Z)-beta-Farnesene	6.45	-	-
	1-Terpinen-4-ol	1.89	-	-
	2-Cyclohexen-1-one, 4-(1-methylethyl)-	0.35	-	-
	3-Carene	0.19	-	-
	3-Thujol	-	0.10	0.28
	4(10)-Thujene	0.12	-	-
	alpha-Bergamotene	0.08	-	-
	alpha-Caryophyllene	0.14	2.47	3.33
	alpha-Phellandrene	0.06	-	-
	alpha-Pinene	0.17	2.88	7.75
	alpha-Terpineol	0.70	0.40	-
	alpha-Terpinolen	-	0.28	-
	alpha-Thujene	0.09	0.11	0.32
	Anisole, p-allyl-	-	0.86	-
	beta-cis-Ocimene	-	0.80	0.06
	beta-Cubebene	1.27	-	-
	beta-Linalool	30.91	-	-
	beta-Myrcene	0.95	1.18	1.22
	beta-Phellandrene	1.81	0.42	0.45
	beta-Pinene	-	2.48	2.48
	beta-trans-Ocimene	-	0.20	0.16
	Borneol	0.44	0.68	1.63
	Bornyl acetate	-	1.74	0.66
	Camphene	0.19	2.47	7.1
	Camphor	0.16	5.39	16.17
	Caryophyllene	5.66	1.50	1.75
	Caryophyllene oxide	-	0.16	-
	cis-beta-Ocimene	2.91	-	-
	cis-Thujone	-	-	34.28
	Copaene	-	0.38	-
	delta-Cadinene	-	0.07	-
	D-Limonene	0.92	1.20	2.29
	Eucalyptol	0.88	5.22	4.94
	Eugenol	-	2.84	-
	gamma-Elemene	-	0.76	-
	gamma-Muurolene	0.09	-	-
	gamma-Terpinene	0.13	0.18	0.19
	Lavandulol	0.85	-	-
	Lavandulyl acetate	4.71	-	-
	Linalool	-	26.27	2.18
	Linalool acetate	28.75	1.54	1.64
	p-Cymene	-	0.28	0.79
	Terpinolene	0.08	-	-
	Thujone	-	14.84	-
	Thujone (stereoisomer)	-	2.43	-
	trans-beta-Ocimene	3.46	-	-
	trans-Thujone	-	-	5.44
	Tricyclo[2.2.1.0(2,6)]heptane, 1,7,7-trimethyl	-	0.07	0.24
	β-Elemene	-	1.11	-
esters	Acetic acid, hexyl ester	0.99	-	-
	Acetic acid, octyl ester	-	0.20	-
	Butanoic acid, hexyl ester	0.31	-	-
	Hexanoic acid, hexyl ester	0.07	-	-
	4-Hexen-1-ol, 5-methyl-2-(1-methylethenyl)-, acetate	-	0.21	0.23
	3-Octanol, acetate	0.18	-	-
	Octen-1-ol, acetate	0.97	-	-
alcohols	1-Octen-3-ol-	0.17	-	-
	3-Octanol	0.43	-	-
	3-Cyclohexen-1-ol, 4-methyl-1-(1-methylethyl)-	-	0.23	0.39
	3-Cyclohexene-1-methanol, alpha, alpha4-trimethyl-	-	-	0.25
	Bicyclo[3.1.0]hexan-2-ol, 2-methyl-5-(1-methylethyl)-, (1.alpha,2.alpha,5.alpha)-	-	-	0.06
ketones	3-Octanone-	1.99	-	-
	Bicyclo[3.1.1]heptan-3-one, 2,6,6-trimethyl-, (1.alpha,2.beta,5.alpha)	-	-	0.10
other	(E,E)-1,3,5-Undecatriene	0.04	-	-
	1,6,10-Dodecatriene, 7,11-dimethyl-3-methylene-, (Z)-	-	0.25	-
	1H-Cycloprop[e]azulene, 1a,2,3,5,6,7,7a,7b-octahydro-1,1,4,7-tetramethyl-, [1aR-(1a.alpha,7alpha,7a.beta, 7b.alpha)]	-	1.12	1.58
	1H-Cycloprop[e]azulene, decahydro-1,1,7-trimethyl-4-methylene-, [1aR-(1a.alpha,4a.alpha,7alpha,7a.beta,7b.alpha)]-	-	0.15	-
	Azulene, 1,2,3,4,5,6,7,8-octahydro-1,4-dimethyl-7-(1-methylethenyl)-, [1S-(1alpha,4alpha,7alpha)]-	-	0.88	-
	Azulene, 1,2,3,5,6,7,8,8a-octahydro-1,4-dimethyl-7-(1-methylethenyl)-, [1S-(1alpha,7alpha,8a.beta)]-	-	1.64	-
	Bicyclo[3.1.1]hept-2-ene, 2,6-dimethyl-6-(4-methyl-3-pentenyl)-	-	4.27	-
non-identified	non-identified	0.47	10.76	2.04

Note: LEO—lavender essential oil; SEO—sage essential oil; BEO—basil essential oil.

**Table 4:** Antibacterial activity of essential oils (MIC, µL/mL) and gentamicin (MIC, mg/mL) by broth microdilution testing.

Samples	*Escherichia coli* ATCC 25922		*Salmonella enteritidis* ATCC 13076		*Staphylococcus aureus* ATCC 6538P		*Listeria monocytogenes* ATCC	
	MIC (μL/mL)	MBC (μL/mL)	MIC (μL/mL)	MBC (μL/mL)	MIC (μL/mL)	MBC (μL/mL)	MIC (μL/mL)	MBC (μL/mL)
LEO	3.795 ± 0.73 ^b^	5.14 ± 0.00 ^b^	5.14 ± 0.00 ^a^	5.14 ± 0.00 ^a^	3.795 ± 0.73 ^a^	5.14 ± 0.00 ^a^	22.68 ± 0.00 ^a^	22.68 ± 0.00 ^a^
SEO	16.74 ± 0.73 ^a^	22.68 ± 0.00 ^a^	5.14 ± 0.00 ^a^	5.14 ± 0.00 ^a^	3.795 ± 0.73 ^a^	5.14 ± 0.00 ^a^	10.80 ± 0.00 ^b^	10.80 ± 0.00 ^b^
BEO	1.17 ± 0.00 ^c^	1.17 ± 0.00 ^c^	2.45 ± 0.00 ^b^	2.45 ± 0.00 ^b^	2.45 ± 0.00 ^b^	2.45 ± 0.00 ^b^	10.80 ± 0.00 ^b^	10.80 ± 0.00 ^b^

Note: Values are expressed as mean of three replicates ± SD. Means with different letters (a–c) within a column indicate significant differences (*p* < 0.05) using Tukey’s honestly significant difference (HSD) test with a confidence interval of 95% or 99%. (LEO—lavender essential oil; SEO—sage essential oil; BEO—basil essential oil).

## Data Availability

The data presented in this study are available in article and Supplementary Material.

## Supplementary Materials

The following supporting information can be downloaded at: https://www.mdpi.com/article/10.3390/metabo12100962/s1, Figure S1: Human colon adenocarcinoma cells (Caco-2) after 24 h of treatment with basil and sage essential oils; Figure S2: Human ovary carcinoma cells (A2780) after 24 h of treatment with lavender and sage essential oils.
