# Supplementary material for: Physicochemical Characterization and Prospecting Biological Activity of Some Authentic Transylvanian Essential Oils: Lavender, Sage and Basil
Source: Metabolites. 2022 Oct 11;12(10):962. doi: 10.3390/metabo12100962 (PMC9607517; doi:10.3390/metabo12100962)
Supplement: Supplementary file 1 [file metabolites-12-00962-s001.zip › metabolites-1943808-supplementary.pdf]

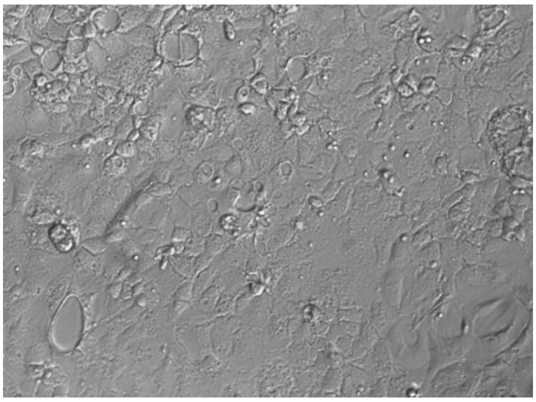

a

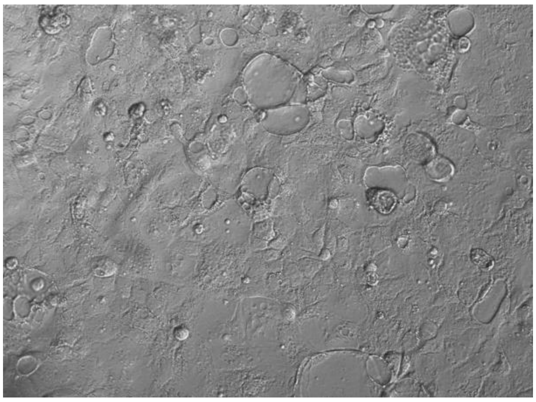

a`

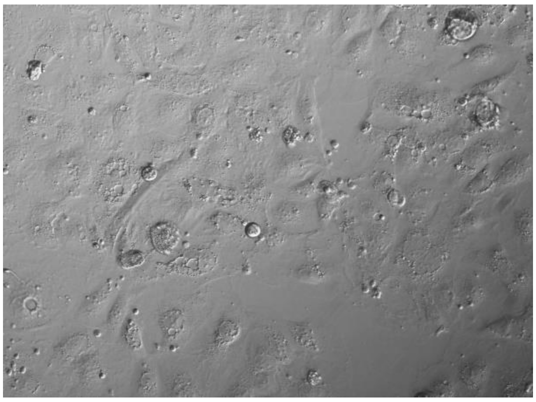

b

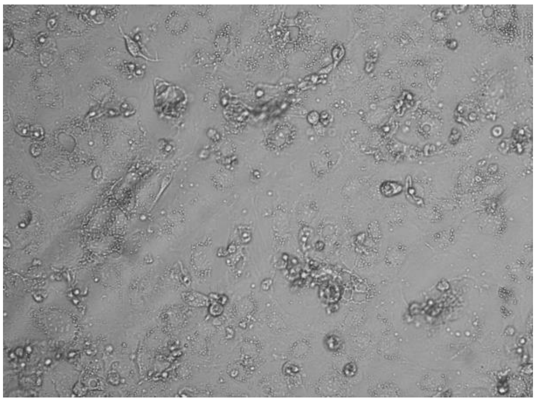

b`

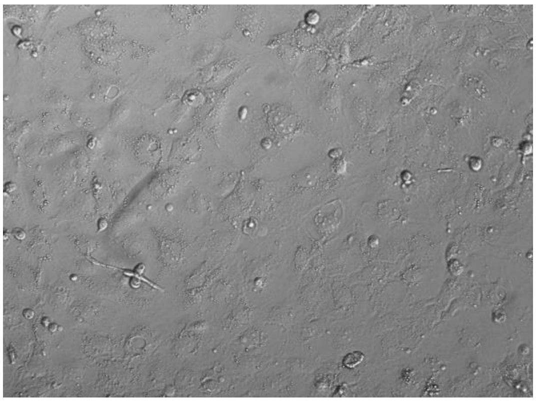

c

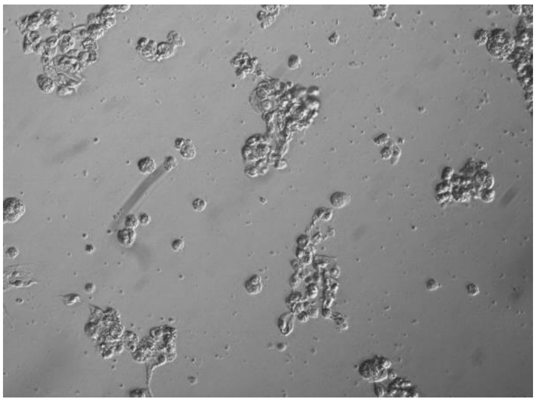

c`

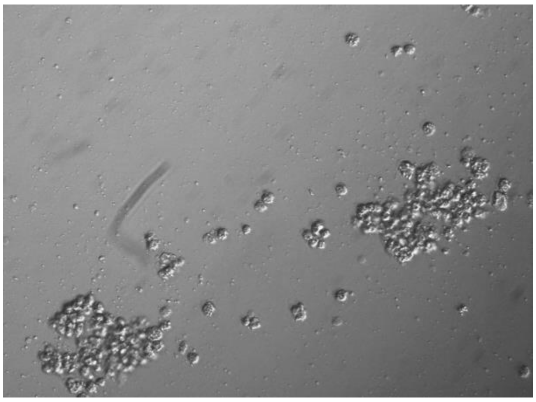

d

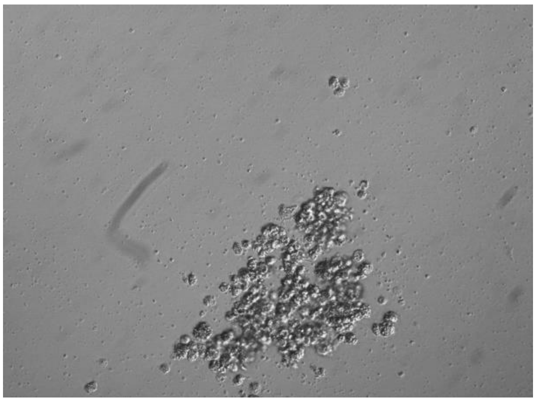

d`

Figure S1. Human colon adenocarcinoma cells Caco-2 after 24 h of treatment with basil and sage essential oils; (a-d – basil essential oil; a`-d` - sage essential oil; a - control; a` - 0.008%; b-b` - 0.016%; c-c` - 0.032%; d-d` - 0.064%).

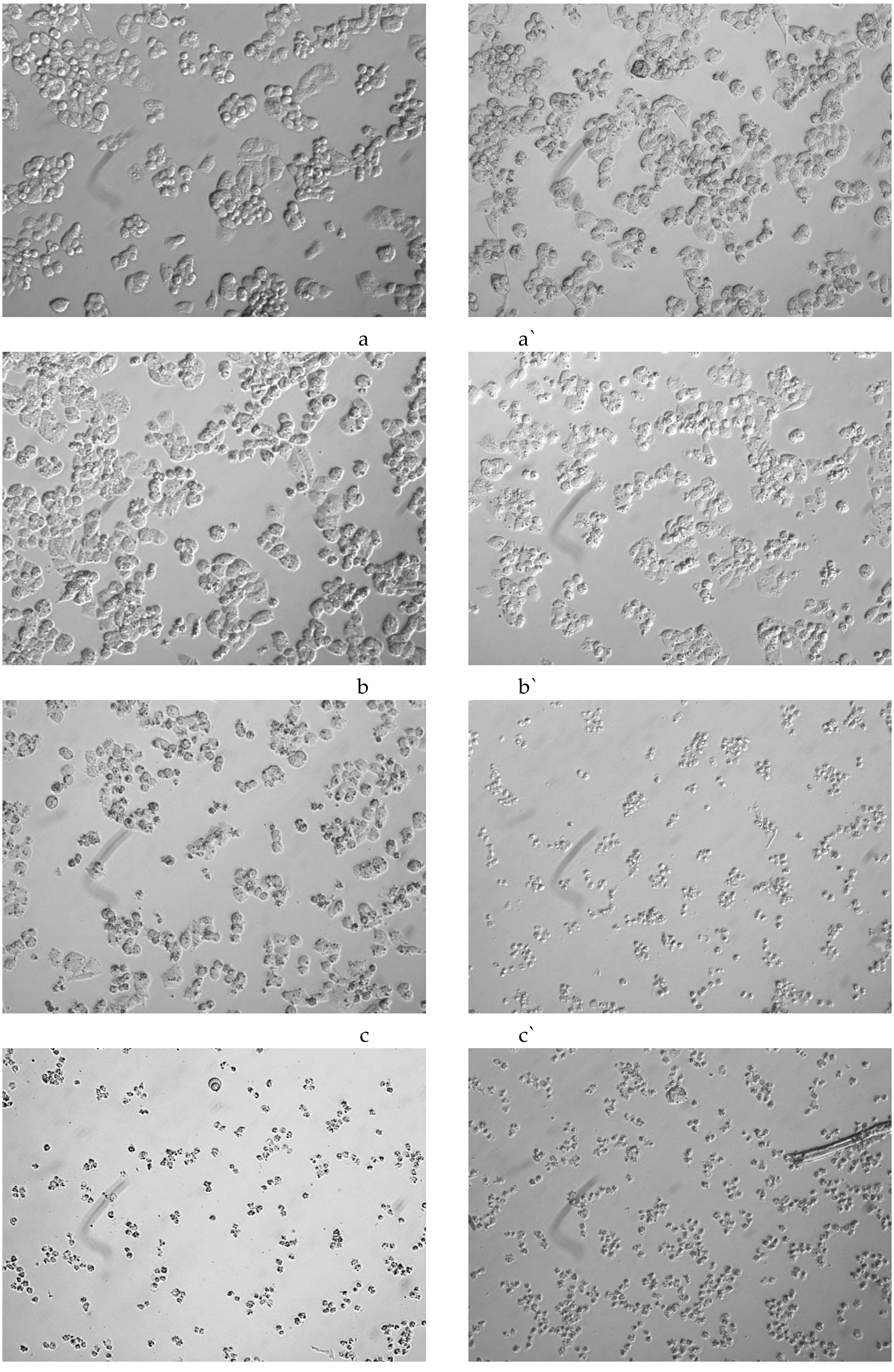

Figure S2. Human ovary carcinoma cells A2780 after 24 h of treatment with lavender and sage essential oils (a-d – lavender essential oil; a`-d` - sage essential oil; a – control; a` - 0.008%; b-b` - 0.016%; c-c` - 0.032%; d-d` - 0.064%).
